# Immediate visual reproduction negatively correlates with brain entropy of parahippocampal gyrus and inferior occipital gyrus in bipolar II disorder adolescents

**DOI:** 10.1186/s12888-023-05012-3

**Published:** 2023-07-18

**Authors:** Haiqin Liu, Weijia Gao, Weifang Cao, Qingmin Meng, Longchun Xu, Liangfeng Kuang, Yongxin Guo, Dong Cui, Jianfeng Qiu, Qing Jiao, Linyan Su, Guangming Lu

**Affiliations:** 1 Department of Radiology, The Second Affiliated Hospital of Shandong First Medical University, Tai’an, China; 2 School of Radiology, Shandong First Medical University & Shandong Academy of Medical Sciences, Tai’an, China; 3 Department of Child Psychology, The Children’ s Hospital, Zhejiang University School of Medicine, Hangzhou, China; 4 Department of interventional radiology, Taian Central Hospital, Tai’an, China; 5 Key Laboratory of Psychiatry and Mental Health of Hunan Province, Mental Health Institute of the Second Xiangya Hospital, Central South University, Changsha, China; 6 Department of Medical Imaging, Jinling Hospital, Clinical School of Medical College, Nanjing University, Nanjing, China

**Keywords:** Bipolar II disorder adolescents, Resting-state functional MRI, Brain entropy, Corticolimbic system, Cortex

## Abstract

**Background:**

Brain entropy reveals complexity and irregularity of brain, and it has been proven to reflect brain complexity alteration in disease states. Previous studies found that bipolar disorder adolescents showed cognitive impairment. The relationship between complexity of brain neural activity and cognition of bipolar II disorder (BD-II) adolescents remains unclear.

**Methods:**

Nineteen BD-II patients (14.63 ±1.57 years old) and seventeen age-gender matched healthy controls (HCs) (14.18 ± 1.51 years old) were enlisted. Entropy values of all voxels of the brain in resting-state functional MRI data were calculated and differences of them between BD-II and HC groups were evaluated. After that, correlation analyses were performed between entropy values of brain regions showing significant entropy differences and clinical indices in BD-II adolescents.

**Results:**

Significant differences were found in scores of immediate visual reproduction subtest (VR-I, p = 0.003) and Stroop color-word test (SCWT-1, p = 0.015; SCWT-2, p = 0.004; SCWT-3, p = 0.003) between the two groups. Compared with HCs, BD-II adolescents showed significant increased brain entropy in right parahippocampal gyrus and right inferior occipital gyrus. Besides, significant negative correlations between brain entropy values of right parahippocampal gyrus, right inferior occipital gyrus and immediate visual reproduction subtest scores were observed in BD-II adolescents.

**Conclusions:**

The findings of the present study suggested that the disrupted function of corticolimbic system is related with cognitive abnormality of BD-II adolescents. And from the perspective temporal dynamics of brain system, the current study, brain entropy may provide available evidences for understanding the underlying neural mechanism in BD-II adolescents.

## Background

Bipolar disorder (BD) is a recurrent mood disorder and usually has its early onset during adolescence. BD is characterized by with high rate of suicidality, comorbid psychiatric and medical problems, and BD is associated with cognitive dysfunction, such as attention, memory and executive function [1], which will lead to a decline in the quality of life. In a latest meta-analysis, the incidence of pediatric bipolar disorder (PBD) was estimated to be approximately 3.9%(2). Bipolar II disorder (BD-II) is featured by a clinical process of recurring emotional episodes, including one or more major depressive episodes and at least one hypomanic episode [3]. BD-II patients have a substantially more chronic course of disease, with significantly more major and minor depressive episodes and shorter intervals between emotional episodes compared with bipolar I disorder (BD-I) [4]. BD-II patients also have cognitive impairment in attention, memory and executive function, especially in adolescents, however, the relationship between impairment of cognition and brain functions remains unclear.

Cognitive dysfunction is one of vital accompanied symptoms in BD patients, particularly in the domains of sustained attention, verbal learning and executive functioning [5]. Previous neuroimaging investigations of adult BD-II patients showed a higher error rate and worse performance on the Stroop color-word test (SCWT) than healthy controls (HCs) [6, 7]. Serum brain-derived neurotrophic factor level was positively correlated with Stroop color naming test in adult BD-II patients which may potentially indicate cognitive dysfunction in BD II patients with a current depressive episode [8]. Visual reproduction subtests include measure of both immediate (visual reproduction I; VR-I) and delayed (visual reproduction II; VR-II). One study conducted VR-I to explore visual-perceptual-motor skills and nonverbal reasoning skills in PBD patients. The scores of VR-I in mania and euthymic PBD groups were significantly lower than that of HCs, and the scores of VR-I in the manic group positively correlated with the volume of right amygdala, bilateral basal ganglia, right lateral nucleus, and right accessory basal nucleus [9]. These findings suggested that the corticolimbic neural circuitry system changes in BD when performing cognitive tasks.

Human brain is a dynamic complex nonlinear system with countless neurons and synapses to transmit internal and external stimuli [10]. Complexity theory may add a new dimension and provide an important method to extract basic features from multiple levels of irregularity and fluctuating neuroimaging data [11]. The complexity of brain dynamic system can be expressed by calculating brain entropy (BEN) of resting-state functional MRI (rs-fMRI) time series [12], because both the time sequence and the space distribution of entropy waves in neuronal elements can have different degrees of order, and brain signals can be considered as entropy waves and the information is coded in the order of these waves [10]. Previous studies demonstrated that significant correlations among BEN, regional homogeneity and amplitude of low-frequency fluctuation (ALFF) were found in schizophrenia and bipolar disorder patients(13, 14). BEN can provide unique characteristics of brain function assessment and neurocognition that are not reflected by ALFF score and cerebral blood flow, besides, BEN was more effective than the functional connectivity method [15]. Altogether, these researches confirmed BEN is a potential biomarker for various brain diseases. An increase in BEN value is associated with more variability, complex dynamics, and higher levels of irregular signals of a system [16].The entropy-based approaches have been widely employed in the study of complexity analysis of rs-fMRI signals in mental disorders, such as schizophrenia, obsessive-compulsive disorder, autism spectrum disorder and BD. Contrast with other parts of the brain, higher entropy was showed in BD patients and its correlations to neurocognition [15, 17]. Few studies had used BEN to assess brain signal complexity of BD patients [15, 18]. Compared to HCs, manic and euthymic adolescent BD (ABD) patients showed increased BEN in the right parahippocampal gyrus (PHG) and left dorsolateral prefrontal lobes, and BEN in the right PHG was associated with the numbers of episode in manic ABD patients. Significantly increased entropy values mainly presented in the middle temporal gyrus, angular gyrus, superior occipital gyrus and medial superior frontal gyrus, and the entropy values of the angular gyrus were significantly negatively correlated with clinical scores in adult BD. Until now, little is known about the brain complexity of ABD, and the conclusions of adult BD cannot be applied to ABD directly. Therefore, the relationship between alteration of brain entropy and cognitive features in BD-II adolescents deserves further study.

The purpose of the current study was to examine the relationship between brain entropy alternation and cognitive features in BD-II adolescents by analyzing the entropy in the rs-fMRI BOLD signals. Firstly, neurocognitive tests including SCWT, VR-I and Digit Span Subtest (DST) were evaluated in BD-II adolescents and HCs. Then, entropy values of all voxels of the brain rs-fMRI data were calculated. Finally, correlation analyses were performed between BEN values and cognitive and clinical indices in BD-II adolescents. Based on previous researches on BD, we hypothesized that some corticolimbic neural circuitry in BD-II adolescents may exhibit higher level of brain entropy that associated with cognitive indicators than that in HCs.

## Methods

### Participants

Thirty-six adolescents, including nineteen BD-II patients and seventeen age-gender matched HCs participated in the current study. All the BD-II adolescents were recruited from psychiatric clinic of the Second Xiangya Hospital of Central South University, and HCs were students recruited from local middle school through advertisement. The data were collected from 2012 to 2014.

Inclusion criteria for BD-II adolescents were: (a) 12–18 years old; (b) having met the diagnostic criteria for bipolar disorder in the Diagnostic and Statistical Manual for Mental Disorders Fourth Edition (DSM-IV); (c) having depressive and hypomanic phases without the full manic episodes, the major depressive episode must last at least 2 weeks and the hypomanic episode must last at least 4 days; (d) right-handedness.

Exclusion criteria for all subjects were: (a) contraindications to MRI scanning, such as claustrophobia, and metallic substance in and out of the body; (b) scores of intelligence quotient (IQ) less than 80; (c) history of head trauma; (d) alcohol or substance abuse; (e) other mental illnesses, including schizophrenia and anxiety disorder; (f) left-handedness; (g) neurological disorders.

### Clinical diagnosis and neuropsychological assessment

All adolescents were accompanied by at least their parents for a diagnostic semi-structured interview independently by two experienced child psychiatrists using Affective Disorders and Schizophrenia for School aged Children Present and Lifetime Versions (K-SADS-PL). diagnostic consistency between the two different child psychiatrists was tested using the inter-rater reliability and yielded satisfactory agreement (Kappa = 0.85).

Demographic and clinical characteristics of all the adolescents were recorded, including name, age, gender, education, age of onset, numbers of episode, course of illness, treatment, family history, the numbers of depression episode, IQ, scores of Young Mania Rating Scale (YMRS) and Mood and Feelings Questionnaire (MFQ). The YMRS is an instrument used to assess the severity of mania in BD patients. The MFQ is a widely used screening measure of depressive symptoms in children aged 8–18 years.

Neuropsychological tests, including Stroop color-word test (SCWT), trail making test (TMT), immediate visual reproduction subtest (VR-I) and digital span test (DST) before MRI scanning, were also recorded.

Stroop color-word test (SCWT) measures the ability to suppress habitual response patterns, working memory and selective attention. The test consists of three parts (SCWT-1, SCWT-2 and SCWT-3), each part has 100 visual stimuli. In the first part (color naming test), subjects were presented a paper with the 100 colorful dots (red, green, blue, and yellow) and asked to pronounce as many of the color of the dots as possible within 45 s. In the second part (word reading test), subjects were presented with a paper with black printed words of ‘red’, ‘green’, ‘blue’ and ‘yellow’, and asked to name as many words as possible within 45 s. In the third part (color word interference test), the subjects were presented a paper with colorful printed of words of ‘red’, ‘green’, ‘blue’ and ‘yellow’, and the color of the words is inconsistent with the meaning of the words (e.g., the word red is printed in blue), the subjects were asked to tell color of the words, rather than the words themselves within 45 s. The correct number of colorful dots, words and color of words were recorded respectively.

Immediate Visual Reproduction Subtest (VR-I) is used to assess visual memory, which check immediate recall and learning rate. Three pictures of geometric figures with increasing complexity are shown in sequence, one at a time. The first and second pictures have one geometric shape, and the third picture has two geometric shapes. During the test, each picture was presented to the subjects for 10s in sequence, and all subjects were required to draw it completely on a paper immediately after viewing each picture. They could modify it at any time during the drawing process but could not modify it again after completing each picture. Points are scored by adding up the correct scores, and the scores for each picture are added to get an overall score. The total score of the test is 15 points and the points are scored according to the drawing of the subjects and the criteria of scoring.

Digit Span Subtest (DST), checking instant auditory memory and attention, consists of two parts (DST-F and DST-B). This test usually selects a series number of different lengths randomly from 1 to 9 as the test material and the digit numbers from less to more. After being shown the stimulus, the subjects were asked to repeat the same number sequence back to the testers in forward order (DST-F) and in reverse order (DST-B). If subjects recite any one item correctly on their first attempt, they can move on to the next item; if there is a mistake, then subjects must try a second time; if the first and second attempts fail, they are not continued and the digit numbers of the previous repeat pair is recorded. The longest number sequence that the subjects could correctly line up was the number memory span of the subjects which is the final score.

### Image acquisition

In the study, a Siemens Trio 3.0 T MRI system (Siemens, Munich, Germany) was used to extract rs-fMRI data. All of the subjects were instructed to lie still, keep awake without thinking anything or falling asleep during scanning. Earplugs were offered to minimize scanning noise and protect children’s hearing, and foam pads were used to limit head motion. Single-shot gradient echo imaging (GRE-EPI) sequence was used to collect rs-fMRI data (repetition time (TR) = 2000ms; echo time (TE) = 30ms; slices = 30; thickness = 4 mm; gap = 0.4 mm; field of view (FOV) = 240 mm × 240 mm; in-plane resolution = 64 × 64 and flip angle (FA) = 90°). T1-weighted images were required by using three-dimensional magnetization-prepared rapid acquisition gradient echo (3D MPRAGE) protocol. (TR = 2300ms, TE = 2.03ms, inversion time = 900ms, thickness = 1 mm, gap = 0 mm, FOV = 256 mm × 256 mm, matrix size = 256 × 256 and FA = 9°)

### Image pre-processing

Data Processing Analysis of Brain Imaging (DPABI V4.2, http://rfmri.org/dpabi) was employed to preprocess the rs-fMRI data with steps as below [1]. The first ten volumes were discarded considering that subjects need time to adapt to the scanning environment. (2) Slice timing was performed to correct for differences in acquisition time between slices [3]. Head motion was adjusted by using the first image as the reference to reduce the effect of head movement during scanning. Subjects with exceeded head motion (more than 2 mm in translation and 2 degrees in rotational motions) would be excluded [4]. T1 images were co-registered to mean rs-fMRI image. The structural images registered with the functional were divided into gray matter, white matter (WM) and cerebrospinal fluid (CSF). The three parts set as masks to extract mean WM and CSF signals of rs-fMRI images and obtain same image resolution as structural images [5]. After head movement correction, the rs-fMRI images were normalized to the Montreal Neurological Institute (MNI) space, and then resampled with 3 × 3 × 3 mm^3^ isotropic voxels [6]. Linear signal drift of rs-fMRI images was eliminated by detrending procedure [7]. WM, CSF signals and Friston 24 head motion parameters were regressed out from fMRI signals as covariables [8] rs-fMRI images were filtered by bandpass filtering (0.01-0.08 Hz) to decrease influences of high frequency physiological noise and low frequent drift [9]. A 6 mm Gaussian kernel of FWHM was used to spatial smooth for rs-fMRI images.

### BEN mapping calculation

The Brain Entropy mapping toolbox (BENtbx, https://cfn.upenn.edu-zewang/ENtbx.php) was employed to conduct brain entropy maps. Sample entropy (SampEn) of time series of each voxel of the preprocessed rs-fMRI were calculated [19]. SampEn, an approximation to Kolmogorov complexity, is employed to quantify the temporal coherence of a time sequence via computing “logarithmic likelihood”. The “logarithmic likelihood” is a small part (within a window of size ‘m’) of the data “matches” with some sections of the data and will still “match” with other sections if the section window size increases by 1. The “match” is decided by a threshold less than product of r and standard deviation of the entire time sequences. Each voxel in the rs-fMRI data is denoted by$x=\left[{x}_{1, }{x}_{2, \dots }{x}_{N }\right]$, where N is the number of a session of rs-fMRI time series. The calculation of SampEn begins with forming a bunch of vectors which were called embedded vectors. Each embedded vector with m continuous time points was extracted from $x$: *u*_*i*_ = [${x}_{i },{x}_{i+1, . . .}{x}_{i+m-1}$], where i= 1 to N− m + 1, and m is the pre-set dimension. ${B}_{i}^{m}\left(r\right)$ is the number of distances less than r from vectors *u*_*j*_ to *u*_*i*,_ and it was calculated by using a pre-defined length threshold r, the above process is repeated to obtain ${B}_{i}^{m+1}\left(r\right)$ of dimension m+1. Then all possible vectors were averaged, we could obtain

1 $${B^m}(\text{r})\frac{1}{(\text{N}-\text{m})(\text{N}-\text{m}-1)} \sum _{\text{i}=1}^{\text{N}-\text{m}}{\text{B}}_{\text{i}}^{\text{m}}\left(\text{r}\right)$$

2 $${A^m} ({r}) = \frac{1}{(\text{N}-\text{m})(\text{N}-\text{m}-1)} \sum _{\text{i}=1}^{\text{N}-\text{m}}{\text{B}}_{\text{i}}^{\text{m}+1}\left(\text{r}\right)$$

And SampEn is computed as:

3 $${SampEn} (m, r, N, x) = -\text{l}\text{n}\left[\frac{{\text{A}}^{\text{m}}\left(\text{r}\right)}{{\text{B}}^{\text{m}}\left(\text{r}\right)}\right]$$

SampEn with different m exhibited similar performance in distinguishing signal from noise, and a smaller m could improve the accuracy of calculation in entropy based on SampEn, so m = 3 was chosen as the empirical value to obtain BEN mapping of whole brain using rs-fMRI data. When r > 0.6, different m produced similar SampEn values and the SampEn difference remained at the same level between the Sinusoidal and chirp signal, therefore, r = 0.6 was chosen as the optimal value for the BEN mapping. In the current study, indices of m and r were set to be 3 and 0.6 separately. A subject’s brain map was obtained by collecting all voxels of BEN values and then smoothed with an isotropic Gaussian kernel (FWHM = 6 mm).

### Statistical analyses

To test whether there were differences in demographic and clinical indices between BD-II adolescents and HCs, categorical variable data were examined by chi-square test, and numeric variable data were tested by two-sample t-test. Partial correlations analysis was conducted to evaluate the relationship between entropy and clinical indices and neuropsychological tests. A p value less than 0.05 is considered to be a significant difference.

Gaussian random field’s (GRF) theory was used to reduce the false positive rate, with voxel level p < 0.005 and cluster level p < 0.05. Clusters less than 137 were removed. Brain areas showing significant differences between the two groups were considered as regions of interest (ROIs) and then mean BEN values of these ROIs were extracted for the further research. As the sample size in each group was relatively small, we have added statistical power (effect size, Cohen’s d) to show differences between the BD-II adolescents and the HCs.

Partial correlations analysis was conducted to evaluate the relationship between entropy and clinical indices and neuropsychological testing scores with gender and age regressed. These residual values were then used to complete the correlation analysis.

## Results

### Demographic and clinical characteristics

All adolescents’ demographic and clinical characteristics were shown in Table 1. No significant difference was found in age, gender and education between BD-II adolescents and HCs. Significant differences were found in scores of immediate visual regeneration subtest, Stroop color-word test, and mood and feelings questionnaire between the two groups.

**Table 1:** Demographic and clinical information of the participants

Characteristics	BD-II (n = 19)	HCs (n = 17)	Statistic (χ2 / T)	p-value	Cohen’s d
Gender (male/female)	7/12	6/11	0.009^#^	0.923	-
Age (years)	14.63 ± 1.57	14.18 ± 1.51	0.884^^^	0.383	0.292
Education (years)	7.68 ± 1.49	7.29 ± 2.02	0.663^^^	0.512	0.220
Medications Lithium	7(37%)				
Valproate	8(42%)				
Atypical antipsychotics	12(63%)				
Antidepressants	3(16%)				
IQ	105.79 ± 13.01	106.00 ± 7.66	-0.058^^^	0.954	-0.020
YMRS scores	8.90 ± 11.09	3.70 ± 2.14	1.895^^^	0.067	0.651
MFQ scores	13.74 ± 10.67	5.59 ± 3.08	3.033^^^	**0.005** ^**^	**1.038**
SCWT-1	52.47 ± 14.90	65.24 ± 12.71	-2.748^^^	**0.015** ^*^	**-0.922**
SCWT-2	69.11 ± 20.72	87.47 ± 8.97	-3.379^^^	**0.004** ^**^	**-1.150**
SCWT-3	29.11 ± 8.71	40.71 ± 9.48	-3.827^^^	**0.003** ^**^	**-1.274**
VR-I	9.11 ± 4.58	13.24 ± 1.52	-3.542^^^	**0.003** ^**^	**-1.210**
DST-B	4.95 ± 2.15	5.65 ± 1.50	-1.121^^^	0.324	-0.378
DST-F	8.68 ± 1.60	8.76 ± 0.75	-0.189^^^	0.851	-0.064

Values are presented as mean ± standard deviation. # Pearson chi-square test; ^Independent-sample t-test. *p < 0.05, **p < 0.01, ***p < 0.001. Presented adjusted p < 0.05 was considered to indicate a significant difference (FDR corrected)

BD-II, bipolar disorder type II; HCs, healthy controls; IQ, intelligence quotient; YMRS, Young Manic Rating scale; MFQ, Mood and Feelings Questionnaire; SCWT, Stroop color-word test; TMT, trail making test; VR-I, visual reproduction immediate recall subtest; DST, digit span test

### BEN analysis

**Fig. 1:**
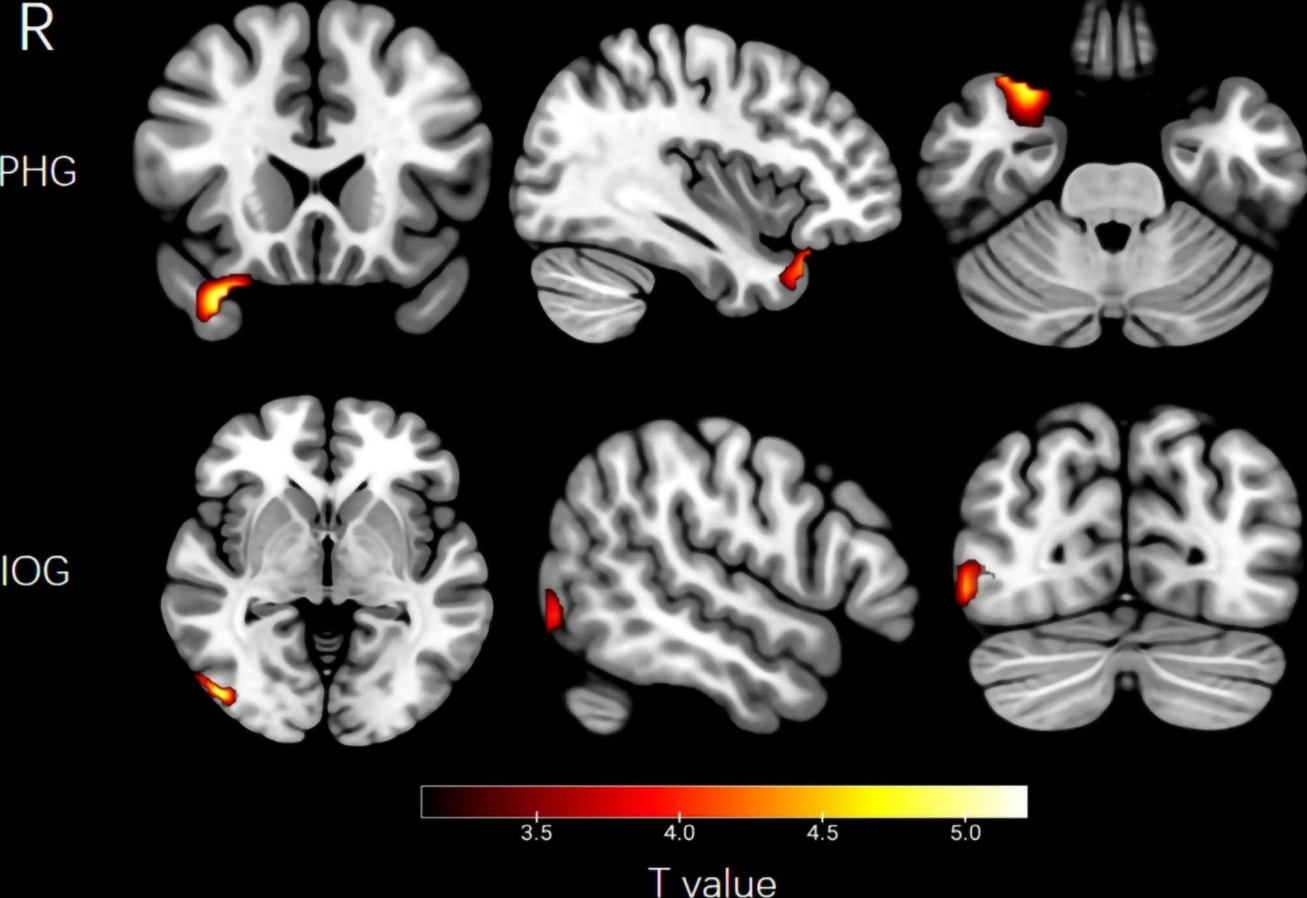
Region of BEN value alteration in BD-II adolescents compared with that of healthy control. Increased BEN values were detected in the right parahippocampal gyrus (PHG) and inferior occipital gyrus (IOG) (GRF correction, voxel level p < 0.005 and cluster level p < 0.05)

Multiple comparisons suggested that compare to HCs, BD-II adolescents indicated increased BEN in right PHG and IOG (GRF correction, voxel level p < 0.005 and cluster level p < 0.05) (see Fig. 1. and Table 2.)

**Table 2:** Brain regions with significant differences in BEN values between BD-II and HCs

Brain regions	Hemisphere	Peak MNI (x, y, z) Coordinates	Cluster size (Voxel)	Peak intensity (T-values)
Parahippocampal gyrus	Right	-30 30 21	111	5.58
Inferior occipital gyrus	Right	-6 45–81	73	5.41

Brain areas showing significant differences between the two groups were considered as regions of interest (ROIs) and then mean BEN values of these ROIs were extracted to obtain statistical analysis value in Table 3.

**Table 3:** Brain regions with significant differences in BEN values between BD-II and HCs

Brain regions	BD-II (n = 19)	HCs (n = 17)	T-value	p-value	Cohen’s d
Right parahippocampal gyrus	326.17 ± 77.79	186.48 ± 93.98	4.877	**< 0.001**	1.619
Right inferior occipital gyrus	379.61 ± 21.07	335.73 ± 25.99	5.590	**< 0.001**	1.854

Values are presented as mean ± standard deviation. p value was FDR corrected

### Correlation analysis

**Fig. 2:**
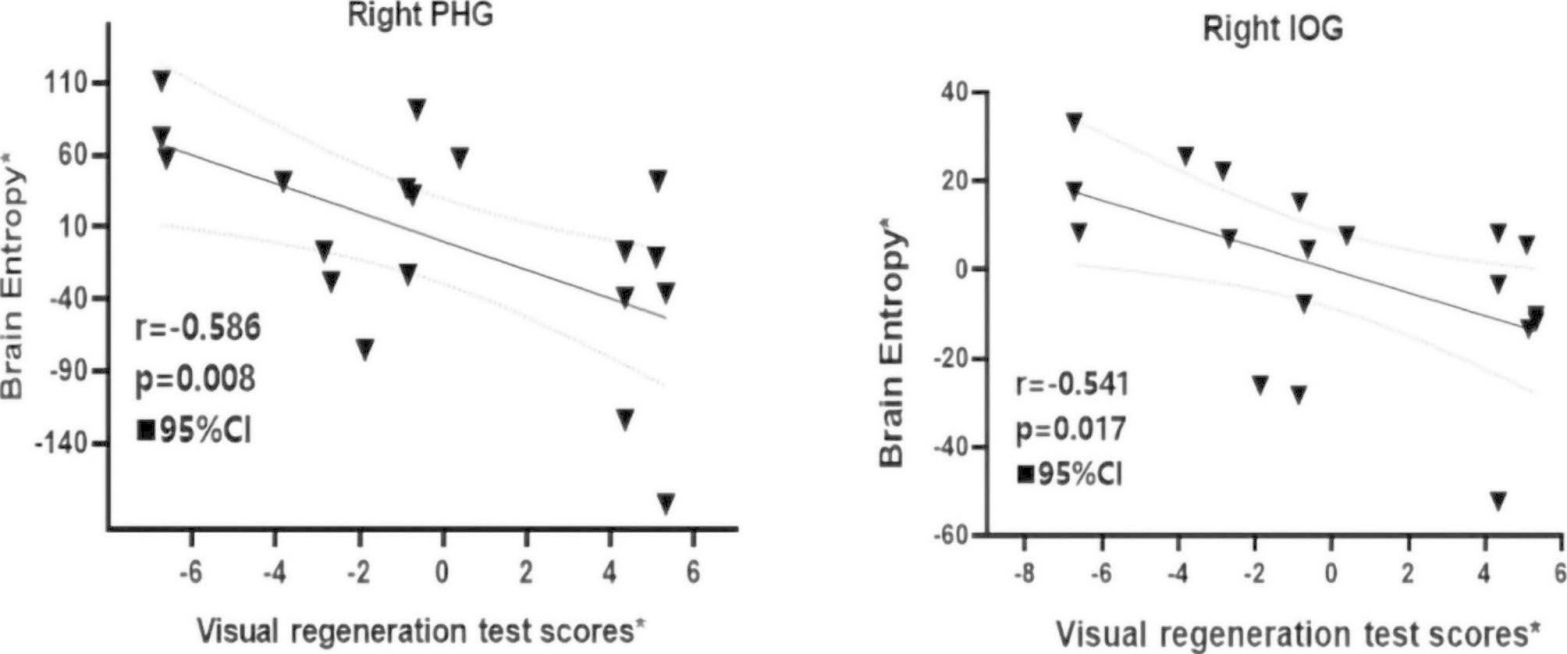
Correlation analysis between entropy values of brain regions and VR-I scores in BD-II adolescents. Entropy values of right parahippocampal gyrus (PHG) and right inferior occipital gyrus (IOG) showed a negative correlation with VR-I scores in BD-II adolescents. * Represents the adjusted values controlling for the influence of the age and gender

Figure 2 showed that values of VR-I were negatively correlated with the entropy of right PHG (r=-0.586, p = 0.008) and right IOG (r=-0.541, p = 0.017) in BD-II adolescents. No remarkable association was found between BEN values and other clinical indicators and neuropsychological tests.

## Discussion

In the current study, neurocognitive functions, and complexity of brain regions with entropy differences were investigated in BD-II adolescents and HCs aged from 12 to 18 years old. Three major findings were as follows: First, significant statistic differences were shown in scores of VR-I, SWCT-1, SWCT-2, SWCT-3; Second, increased BEN values were found in right PHG and right IOG in BD-II adolescents compared with that of the HCs; Third, BEN values of right PHG and right IOG exhibited significantly negative relationship with scores of VR-I in BD-II adolescents.

We tested the hypothesis that neuropsychological impairment in sustained attention, verbal learning and executive functions were also presented in BD-II adolescents, which consisted with neurodevelopmental dysfunction in adult BD-II patients. In the three parts of SCWT, color naming test predicts working memory and speed of visual search, word reading test responds speed of visual search, and color word interference test reflects both conflict monitoring and working memory [20] of the subjects. In the present study, scores of all the three parts of SCWT in BD-II adolescents were significantly lower than that of HCs, suggesting impairment of these cognitive functions occurred in BD-II adolescents. It had been reported that young stabilized and adult type II bipolar patients performed significantly worse than controls in the SCWT, mainly on color word interference test of SCWT, which was the most significant endophenotype of BD [6, 7]. These findings were consistent with the current study. Considering the involvement of lingual gyrus in color processing, extrastriate cortex in visual word-form processing of word reading, and ventral prefrontal in color word interference [21], the decrease of SCWT scores may indicate disturbances of BD-related subcortical-cortical functional coupling in BD-II adolescents. VR-I was conducted to assess the visualspatial memory of the subjects in the present study, with the significantly reduced scores of the BD-II patients than that of the HCs. The test of VR-I is involved in visual-perceptual-motor skills and nonverbal reasoning skills. In VR-I, there are four geometric pictures that composed of flags, and a factor analysis showed that flags were a more unique measure of visual memory [22]. The performance of VR-I is associated with other cognitive functions. Executive impairments specifically affected visual learning, encoding and retrieval and only executive tests with visuospatial components can predict recall of visual reproduction stimuli [23]. VR-I had been regarded as excellent sensitivity of cognitive functioning rather than as specific measures of memory in a study of Alzheimer disease (AD) [24]. VR-I has also been used to explore cognitive disruption in psychological illnesses, such as schizophrenic and PBD. A significant lower score has been found on the VR-I in mania and euthymia PBD groups [9]. And schizophrenic patients recalled less information than HCs [25]. The findings suggested that these cognitive impairments (visualspatial memory/processing speed and self-regulation/monitoring) also presented in the early onset (adolescent onset) or early stage of BD-II adolescents.

In the present study, BD-II adolescents exhibited significant increase of BEN values in right PHG and right IOG, which meant more variability, complex dynamics, and higher levels of irregular signals of the two brain regions in BD-II adolescents. PHG locating in the medial temporal lobe and constituting a large portion of the limbic lobe, is a highly ordered cortical region that deliver information over long distances from one area of gray matter in the cortex to another [26]. A study has demonstrated PHG is thought to be related to episodic memory, emotional and visuospatial processes [27], more specifically, there was evidence suggesting that associative contextual memory retrieval, scene perception and spatial representation [28] are supported by the PHG. In the BD-related corticolimbic model, PHG is involved in the ventromedial circuit and directly connected to the subgenual prefrontal cortex. The ventromedial circuitry is account for the control of internal emotions, thus, it can regulate the emotional states generated internally [29]. Structural and functional abnormalities of PHG have been demonstrated in BD patients, including decreased volume in PBD patients with psychotic symptoms [30] and manic, euthymic PBD groups [31]. Adult BD-II patients exhibited increased functional connectivity strength and greater resting metabolic rates in the bilateral PHG [32]. A recent paper [18] has reported increased BEN in right PHG in manic and euthymic BD adolescents. Taken together, the increased BEN in PHG found in the present study may further illustrate disrupted complexity of BOLD signals of the ventromedial circuit which was related to internal emotional control. At this point, in a recently published paper [33] has found that complexity of electroencephalogram (EEG) signal increased in a period of brain development, and it may be related to enhanced arborization of dendritic trees [34], axons [35] and synaptic stabilization [36] that were associated with cytoarchitectural or synaptic functions. During adolescence, rapid and concurrent neural changes of brain development and synaptic remodeling are presented [37] when two major dynamic processes take place in mature brain: synaptic pruning [38] and myelination. Synaptic pruning is a process of synapse elimination that is involved in both regulation of needed synaptic connections and terminal pruning of learning and memory [39]. Eastwood et al. [40]demonstrated that decreased expression of two presynaptic proteins (complexin I and II) in inhibitory and excitatory neurons in the parahippocampal subregion in adult BD patients, which may indicate abnormalities in cellular structure or synapses. Therefore, increased PHG entropy of BD-II adolescents may be attributable to neuronal and synaptic abnormalities in PHG.

IOG is the most posterior brain region and one of core human neural systems for face perception, which is associated with initial stage of face processing, specifically, the processing of facial features [41]. And gamma oscillations of right IOG are related to the early stages of eye processing [42]. The inferior occipital gyrus has been implicated as an important region for emotion regulation in mood disorders [43]. One longitudinal study of healthy children and adolescents indicated that the process of occipital cortex loss began around puberty [44], which indicated that occipital volume is reduced at the onset of the disease and increases with age. Resting state functional connectivity between the left cuneus and the left IOG was increased in euthymic PBD patients compared with HCs [45] and a significantly decreased ALFF was reported in bilateral IOG of the manic PBD patients [46]. Zhang et al. [47] suggested that response pattern of inferior occipital gyrus dullard was a general failure to identify regulatory needs in adult BD-II patients. The altered BEN in IOG might be associated with abnormal emotional regulation. Adult BD patients demonstrated lower mean multiscale sample entropy values of gray matter than HCs in IOG [13],which was inconsistent with the findings of the current study. The contradictory results of decreased and increased complexity may be associated with discrepancies in the indexes of the selected analysis procedure, diversity of analyzed signals [15], differences in the phase of illness studied, age, medications, and illness severity [48]. In an information-theoretic rendition, entropy is the long-term average of surprisal, and reducing information-theoretic free energy amounts to improving the world model so as to reduce prediction errors, hence reducing surprisal. Thus, it may be the case that BD-II adolescents are less able to form accurate predictions and demonstrate increased entropy earlier on in life [49].

Finally, we examined between BEN values and scores of neuropsychological tests in our entire cohort of BD-II adolescents after controlling for the effect of age and gender. BEN value of right PHG and IOG exhibited significantly negative relationship with scores of VR-I in BD-II adolescents. The finding indicated that increase of BEN of right PHG and right IOG was accompanied by decreased scores of VR-I in BD-II adolescents. It has been reported that an increase in brain complexity in BD patients assessed from EEG signals by Higuchi’s algorithm is associated with a decrease in long-range phase synchronization [50] and neural synchronization [44]. Phase synchronization is a direct marker of enhanced interaction between two brain regions, and neural synchronization in the visual cortex plays a role in the combination of different but related visual features, so as to recognize a complete visual pattern as a whole through animal experiments [51]. This specific correlation might suggest greater randomness in the recruitment of brain areas as a function of visual-perceptual-motor and nonverbal reasoning skills in BD-II adolescents, and lead to a more thorough understanding of the relationship between brain function and neuropsychological characteristics of BD-II adolescents. Previous study demonstrated that lower scores in VR-I were associated with decreased volume of right amygdala, bilateral basal ganglia, right lateral nucleus and right accessory basal nucleus in PBD patients [9]. Negative correlation between BEN of right PHG, right IOG and scores of VR-I in BD-II adolescents further suggested that changes of neural firing patterns and neuronal synchronization in the two brain regions which associated with visual memory may affect the visual memory, immediate recall and learning rate that represented by VR-I. The results expanded our understanding of neural activity abnormalities in BD-II adolescents.

Three limitations in the current study needed to be considered. First, due to the small sample size of adolescent BD-II group not representative of all BD-II adolescents, more data is needed to ensure the sensitivity and presumption of these results. Second, as our study is a cross-sectional experimental design research, longitudinal design studies are needed to increase the understanding of the dynamic changes of brain complexity during the development of BD-II adolescents. Third, in this study, nearly half of the BD-II adolescents were taking medication when MRI scanning, thus, medication exposure becomes a potential influence in the study. However, previous studies have reported that medication appeared to influence many structural MRI results, but had limited influences on functional MRI [52, 53]. And no significant impact of medication in behavioral [54] and neural differences [55] between BD patients and HCs. The medication appeared to normalize brain function in PBD patients. Therefore, effects of medication are unlikely to explain our results as we found increased brain entropy which may be related to neural abnormalities. In future studies, more subjects without taking medication could be recruited to eliminate the effects of the medications.

## Conclusions

Higher entropy in right PHG and right IOG in BD-II adolescents may lead to dysfunction of corticolimbic circuitry which is critically important to emotional processing and cognitive control. The negative correlation between entropy of right PHG, right IOG and scores of VR-I in BD-II adolescents further suggested that the brain complexity was closely related to clinical symptoms. From the perspective of brain temporal dynamics, the finding complements previous studies that corticolimbic dysfunction is an important pathophysiological mechanism of PBD. Brain entropy may provide available evidence for understanding the underlying mechanism in BD-II adolescents.

## Data Availability

The dataset supporting the conclusions of this article is included within the article.

## List of abbreviations

BEN: Brain entropy
BD: Bipolar disorder
BD-II: Bipolar II disorder
BD-I: Bipolar I disorder
HCs: Healthy controls
PBD: Pediatric bipolar disorder
rs-fMRI: Resting-state functional magnetic resonance
ALFF: Amplitude of low-frequency fluctuation
ABD: Adolescent BD
PHG: Parahippocampal gyrus
IOG: Inferior occipital gyrus
VR-I: Immediate visual reproduction subtest
VR-II: Delayed visual reproduction subtest
SCWT: Stroop color-word test
TMT: Trail making test
IQ: Intelligence quotient
YMRS: Young Manic Rating scale
MFQ: Mood and Feelings Questionnaire
DST: Digit span test
